# Editorial: Role of Sex Steroids and Their Receptor in Cancers

**DOI:** 10.3389/fendo.2022.883229

**Published:** 2022-04-06

**Authors:** Pia Giovannelli, Pandurangan Ramaraj, Cecilia Williams

**Affiliations:** ^1^Department of Precision Medicine, University of Campania “L.Vanvitelli”, Naples, Italy; ^2^Department of Biochemistry, Kirksville College of Osteopathic Medicine, A.T. Still University, Kirksville, MO, United States; ^3^Department of Protein Science, SciLifeLab, KTH Royal Institute of Technology, Stockholm, Sweden

**Keywords:** cancers, sex steroid receptors, progesterone receptor (PR), androgen receptor (AR), estrogen receptor (ER), glucocorticoid receptor

The way we view steroid hormones has changed overtime: from simple transcription factors targeting male and female sexual organs, such as epididymis and testes or breast, ovary and uterus, respectively, to complex signalling proteins able to regulate a plethora of processes in a wide range of cell and tissues. Sex steroid receptors were classically considered transcription factors controlling a variety of responses in reproductive tissues both at physiological and at pathological level. Principally represented by oestrogen, progesterone, androgen, and glucocorticoid receptors (ER, PR, AR, and GR), upon binding their hormone, they translocate to the nucleus where recognize specific hormone responsive elements (HREs) located by the promoter of different genes and regulate their transcription (1). In more recent times, numerous studies have demonstrated that steroid receptors also can work in a non-transcriptional manner (2). In a few seconds or minutes after ligand binding, sex steroid receptors activate transduction pathways (such as PI3K/AKT or MAPKs) and alter a multitude of physiological and pathological processes not only in organs recognized as steroid-dependent but also in distinct anatomical sites. By both “genomic” and “non-genomic” mechanisms, steroid receptors influence the regulation of key genes, important for organ development and function but also promote the development and the progression of cancers by influencing tumour growth and invasiveness, epithelial-mesenchymal transition (EMT; 2–4).

In addition to the classical hormone-related cancers of the breast, prostate, ovary, and testis, an increasing number of scientists is studying the role of sex steroid receptors in different kind of cancers (5–14), trying to understand how and when steroid hormones and their receptors influence their incidence in men or women (5, 15, 16).

This Research Topic focuses the attention on the role of steroid receptors in all types of cancers and highlights the importance of updating detection methods to include all isoforms and variants that are continuously discovered. To date, at least 20 different variants of the androgen receptor in prostate (17), 5 variants for the oestrogen receptor β (named from ERβ1 to ERβ5) and 3 variants for the oestrogen receptor a (the full ERα, and two truncated forms ERα36 and ERα46) have been characterized. Pagano et al. illustrate the importance of the newly discovered ERα variant, ERα36, in different human cancers. This variant, with a molecular mass of 36kDa, is involved in tumour progression, metastatic potential, drug-resistance and is expressed in a wide range of human cancers such as neuronal tumours, gastric cancer, hepatocarcinoma, laryngeal, endometrial, renal cell, and papillary thyroid carcinomas. Its expression is also revealed in ER-positive and ER-negative breast cancers where it could be responsible for the drug-resistance.

It’s equally important to choose the best model and use the right technique to study the role of steroid receptors in cancer, as demonstrated by Lacouture et al. By using a FACS-free method, they isolate ERα-positive mammary mouse epithelial cells that, in 3D cultures completely recapitulate the mammary gland’s morphology. In their study, the authors highlight the role of estrogen or ERα in controlling mammary gland metabolism during carcinogenesis. The expression of steroid receptors in classically hormone-dependent cancers has long been used to select the more efficient therapy, but upcoming studies have tried to analyse their involvement in predicting other clinical and biological features of cancers such as overall and disease-free survival, therapy responsiveness, and prognosis. For example, in metastatic breast cancer patients, the prognosis of single hormone receptor (ERα or PR) positive tumours, with or without the HER2 overexpression, was similar as that of double-positive or double-negative (ERα and PR) tumours, indicating that other characteristics, such as age and race of patients, tumour grade, TNM stage, and surgery, have a major weight (Mao et al.). Another important marker for breast cancer is the AR. Its expression is, in most cases, a good prognostic factor in ERα-positive breast cancer and a poor prognostic factor in ERα-negative breast cancer (18, 19). In post-menopausal women, the AR expression is associated to a better survival outcome, while high levels of circulating androgens and an high AR/ER ratio are associated with poor outcomes in ERα-positive breast cancer (18, Rajarajan et al.). Rajarajan et al. evaluated the AR/ER ratio in pre–menopausal breast cancer patients and observed that, also in women younger than 50 years old, a high AR/ER ratio was a poor prognostic factor. They concluded that is not exclusively the AR expression, but the ER activity and the hormonal milieu that determine the clinical outcome. In addition to steroid receptors, Ki67, a proliferation marker, can be used to indicate the responsiveness to neoadjuvant endocrine therapy in ERα–positive breast cancer (Zhang et al.).

The major novelty of this Research Topic lies in the assembled data covering the role of steroid receptors in cancers not viewed as hormone responsive. Different research groups enabled this issue by submitting review and original articles. Bernardo et al. described that, in bladder cancer, besides to the GATA3 expression, higher in low grade and low stage tumours, the ERα expression is lower in low grade tumours, but the reduced number of cases makes it difficult to define the prognostic role of ERα or ERβ in these cancers. Wang et al. demonstrated that in oesophageal cancer, oestradiol inhibits cell viability and migration, thereby providing a novel insight for cancer development, treatment, and prevention. These data justify the sex difference observed in the occurrence of this group of cancer. In glioblastoma, PR and the cytoplasmic kinase src work together to regulate the activity of proteins, such as the focal adhesion kinase (FAK) and paxillin, involved in migration and invasion. Furthermore, the c–src activation could be responsible for the putative PR phosphorylation on Y87 residue, thus connecting genomic and non–genomic action triggered by progesterone, as studied by Bello–Alvarez et al. Indukuri et al. underlined that in the colon, the ERβ influences the inflammatory signalling through NFκB possibly reducing the incidence of colorectal cancers. In particular, by comparing two different colon cancer–derived cell lines, and adding expression of ERβ, they observed that the steroid receptor hinders p65 chromatin binding to genes controlling cell adhesion, migration, and circadian clock, while enabling binding by genes modulating cell proliferation and Notch signalling.

All the collected manuscripts indicate that a deepened knowledge of steroid hormone receptors could help the precision medicine to predict the impact of gender on tumours’ incidence and help developing personalized therapies to efficaciously cure a wide group of cancers.

## Author Contributions

All authors listed have equally, substantially, and intellectually contributed to this editorial and approved it for publication.

## Funding

The authors acknowledge funding by the Swedish Cancer Society (grant no. 21 1632 Pj), and Region Stockholm (HMT, grant no. RS 2021-0316) and Valere Program (Vanvitelli per la Ricerca Program).

## Conflict of Interest

The authors declare that the research was conducted in the absence of any commercial or financial relationships that could be construed as a potential conflict of interest.

## Publisher’s Note

All claims expressed in this article are solely those of the authors and do not necessarily represent those of their affiliated organizations, or those of the publisher, the editors and the reviewers. Any product that may be evaluated in this article, or claim that may be made by its manufacturer, is not guaranteed or endorsed by the publisher.
